# The oncobiome; what, so what, now what?

**DOI:** 10.20517/mrr.2024.89

**Published:** 2025-02-27

**Authors:** Munawar Abbas, Mark Tangney

**Affiliations:** ^1^APC Microbiome Ireland, University College Cork, Cork, T12 YT20, Ireland.; ^2^Cancer Research@UCC, University College Cork, Cork, T12 XF62, Ireland.

**Keywords:** Microbiome, cancer, tumor microenvironment, cancer therapy, DNA

## Abstract

Microbial communities inhabiting various body sites play critical roles in the initiation, progression, and treatment of cancer. The gut microbiota, a highly diverse microbial ecosystem, interacts with immune cells to modulate inflammation and immune surveillance, influencing cancer risk and therapeutic outcomes. Local tissue microbiota may impact the transition from premalignant states to malignancy. Characterization of the intratumoral microbiota increasingly reveals distinct microbiomes that may influence tumor growth, immune responses, and treatment efficacy. Various bacteria species have been reported to modulate cancer therapies through mechanisms such as altering drug metabolism and shaping the tumor microenvironment (TME). For instance, gut or intratumoral bacterial enzymatic activity can convert prodrugs into active forms, enhancing therapeutic effects or, conversely, inactivating small-molecule chemotherapeutics. Specific bacterial species have also been linked to improved responses to immunotherapy, underscoring the microbiome’s role in treatment outcomes. Furthermore, unique microbial signatures in cancer patients, compared with healthy individuals, demonstrate the diagnostic potential of microbiota. Beyond the gut, tumor-associated and local microbiomes also affect therapy by influencing inflammation, tumor progression, and drug resistance. This review explores the multifaceted relationships between microbiomes and cancer, focusing on their roles in modulating the TME, immune activation, and treatment efficacy. The diagnostic and therapeutic potential of bacterial members of microbiota represents a promising avenue for advancing precision oncology and improving patient outcomes. By leveraging microbial biomarkers and interventions, new strategies can be developed to optimize cancer diagnosis and treatment.

## INTRODUCTION

**Figure 1:**
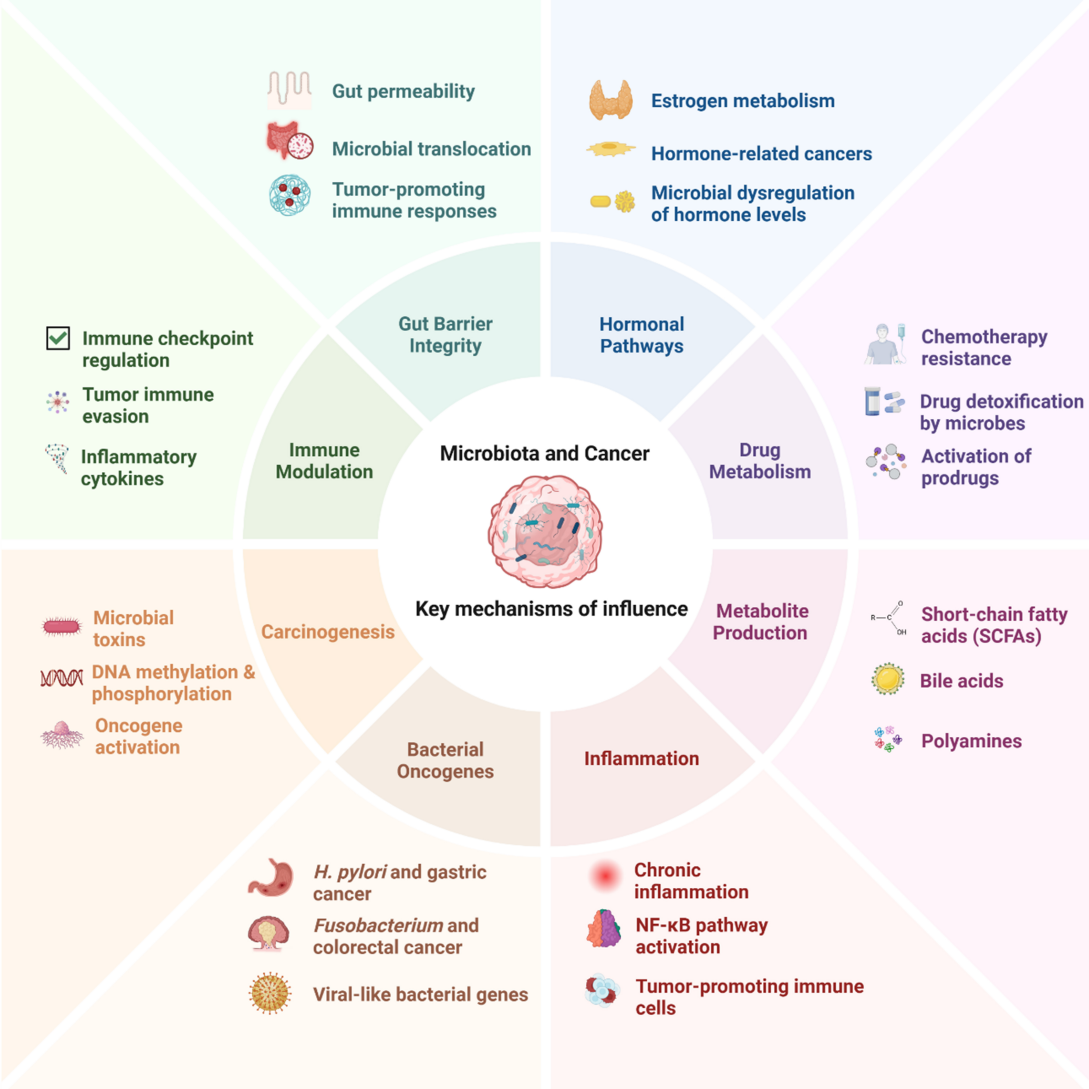
Microbiota’s key mechanisms to influence cancers. Each mechanism details microbiome-related factors, and these pathways highlight the complex interactions between the microbiome and cancer development or treatment responses.

Cancer is one of the leading causes of morbidity and mortality worldwide. The International Agency for Research on Cancer (IARC) reported ~20 million new cases of cancer in 2022 alongside 9.7 million deaths^[1]^. This number, by 2030, is estimated to reach 24 million annually^[2]^. Besides various factors influencing cancer, microbiota, particularly bacteria, have gained significant attention over the previous decade. The term microbiome, as Berg *et al.* have previously reviewed, is not limited to the community of microorganisms but also includes their “theatre of activity”^[3]^. Host and microbiota together form a complex “organism” in which a symbiotic relationship confers health effects on the host. However, defects in the regulation of bacterial sensing and homeostasis by the host, and environmentally induced changes in the microbiome, may disturb this symbiosis and promote diseases such as cancer^[4]^. Increasing evidence indicates a key role of bacterial microbiota in cancer [Figure 1]^[5,6]^.

## WHAT?

The “oncobiome” refers to the collection of microorganisms, including bacteria, viruses, fungi, and other microbes, that are associated with cancer development, progression, and treatment response^[7]^. Therefore, the intricate relationship between cancer and microbiota has introduced the idea of “oncobiosis”, i.e., the imbalance in microbial ecology in the presence of neoplasia^[8]^ (What). This imbalance has been reported to influence the genesis, progression, and treatment efficacy of cancer (So What). For example, a high abundance of *Fusobacterium nucleatum* is known to play a pro-tumorigenic role in colorectal cancer (CRC)^[9,10]^. Bacterial dysbiosis is not only limited to local tissue, but extends to other compartments such as the gut and the bloodstream^[11]^. The microbiome of each body part has distinctive characteristics regarding population dynamics and the diversity of microbial species^[12]^.

Gut residents play a fundamental role in modulating host immunity, both innate and adaptive immune responses^[13,14]^, critical for malignancy control or progression. Certain bacterial species and their metabolites have been reported to improve patients’ immune ability to destroy tumor cells. For example, short-chain fatty acids (SCFAs), butyrate in particular, produced by gut bacteria like *Faecalibacterium prausnitzii*, have anti-inflammatory effects that can suppress tumor-promoting inflammation in the colon^[15]^. Conversely, pathogenic bacteria, through the release of toxins and inflammatory cytokines, can create a pro-tumorigenic environment^[16]^. This dual role of bacteria, in both promoting and preventing cancer, highlights the complexity of the microbiome’s involvement in oncogenesis and the potential for microbiome-based therapies to either enhance anti-cancer immunity or alleviate pro-cancer inflammation. Though it has been estimated that there are trillions of organisms in the human microbiome, IARC currently designates only 11 as being directly carcinogenic (Group 1 carcinogens) to humans. These 11 organisms include one species of bacteria (*Heliobacter pylori*), seven species of viruses, and three species of parasitic worms, which together are responsible for about 2.2 million cancer cases annually worldwide^[17]^. The field of microbiome research is rapidly evolving, and several research groups worldwide are reporting the associations of several bacterial candidates with the incidence of cancer and treatment efficacies. This review highlights the current knowledge and future directions in the study of the bacterial microbiome in relation to cancer.

### Relevance of microbiome to cancer

#### Gut microbiome

The mammalian gut is one of the most complex communities with trillions of microbes including bacteria, archaea, fungi, and viruses^[18]^. Gut microbiota, bacteria in particular, play a significant role in preventing disease and can influence health by metabolizing nutrients, producing metabolites, maintaining the integrity of the mucosal barriers, and developing a healthy immune system. In general, high gut microbial diversity is recognized as a good health indicator^[19]^. This importance of diversity has been shown during the recent COVID pandemic, where Ward *et al.* showed the association between the presence of *Porphyromonas endodontalis* (oral) and *Enterococcus faecalis* (gut) and the severity of COVID infections^[20]^. Similarly, *Ruminococcus gnavus* has been shown to be directly associated with COVID infection severity and is known as an inflammatory marker^[21]^.

Growing evidence suggests the role of the gut microbiome in disease. Compositional and functional changes in the gut microbiome have been linked to a number of diseases, including obesity, diabetes, cardiovascular diseases, and several types of cancers. The gut microbiota is intricately linked to digestive cancers, including gastric cancer, CRC, and liver cancer. Particularly, the presence (or abundant presence) of certain bacteria correlates with an increased incidence of cancer^[22]^. Tumor development is greatly dependent on the nature of the local and systemic immune activity, and the gut microbiome plays a key role in immune modulation^[23]^. Thus, disruption in gut microbiota (dysbiosis) is linked to the initiation and progression of several cancer types such as gastric, colorectal, and liver cancers. For example, the presence of *H. pylori* is known to promote gastric cancer by inducing chronic inflammation resulting in DNA damage^[24-26]^. A number of genes present in this bacterium disrupt cell homeostasis, resulting in the accumulation of cytokines and other cancer-related signaling molecules in infected individuals, leading to gastric cancer. *H. pylori* can also cause esophageal cancer^[27,28]^. The distal part of the esophagus is densely populated by Gram-positive bacteria such as Firmicutes^[29]^. Dysbiosis happens to be advantageous for Gram-negatives as they outnumber the Gram-positives, which can later result in esophagitis. Bacteroidetes, Firmicutes, and Proteobacteria are the dominant esophageal cancer-linked phyla^[30]^.

The large intestine contains 10-fold more gut microbial population than the small intestine, and local GIT cancer incidence follows this. Large intestine residents produce large quantities of metabolites with both cancerous and anti-cancer properties^[31]^. Several studies have shown the relevance of certain gut bacteria to CRC, e.g., *Fusobacterium nucleatum*, *Bacteroides fragilis*, *Clostridium septicum*, *E. coli*, and *Enterococcus faecalis*. These bacteria are known for promoting tumorigenesis through immune evasion and pro-inflammatory signaling, such as the activation of β-catenin pathways^[32,33]^*.* Another possible contribution to CRC could be the diminished population of butyrate-producing species^[34]^. Several mouse studies have reported increased tumor growth in dysbiotic gut models, possibly as a result of inflammation. Meanwhile, reduced incidence of colitis and colon cancer is reported in germ-free mice. For example, Liu *et al.* reported the diminished incidence of colitis and colon cancer in IL-10 knocked out germ-free mice, suggesting the role of inflammation in cancer incidence^[35]^.

Several preclinical and clinical studies have reported the association between gut microbiota alteration and diseases like obesity and liver cancer^[36-38]^. In obese individuals, the risk of epithelial damage is increased, which may allow the commensals to enter the bloodstream. Consequently, this gut microbial dysbiosis may lead to obesity-related liver carcinomas^[22]^. Gut microbial dysbiosis is associated with various liver-related conditions, including cirrhosis, alcoholic- and non-alcoholic fatty liver diseases, and liver cancer. In cirrhosis patients, liver cancer accounts for the most frequent and prominent cause of death^[39,40]^. Gut microbiota produces secondary metabolites that keep the immune system downregulated in the liver, including bile acids and lipopolysaccharides. Bile acids serve as a bridge between gut bacteria, the liver, and the intestine^[41]^. These acids act as emulsifying agents for lipids, cholesterols, and lipid-soluble vitamins. Certain gut bacteria such as *Clostridium hiranonis*, *C. hylemonae*, and *C. scindens* convert primary bile acids to secondary acids^[42]^. Studies have shown that high levels of secondary bile acids produce reactive oxygen species (ROS) and reactive nitrogen species (NOS) that ultimately result in DNA damage, leading to colon cancer^[43]^. In addition, Toll-like receptor-4 (TLR-4) activated by lipopolysaccharides promotes the development of liver cancer^[44]^, suggesting the influential role of the gut microbiome in liver and colon cancer.

Beyond the digestive system, gut microbiota also influences extraintestinal tumors. Recent studies highlight their role in breast and prostate cancer, as well as gliomas and other non-digestive malignancies^[45]^. Breast cancer is one of the leading causes of death in women, accounting for over 685,000 deaths in 2020 alone^[46]^. In addition to genetics and diet, microbiota and their metabolites have been speculated to be one of the factors contributing to the incidence of breast cancer^[47]^. The gut microbiota regulates functions and processes in nearly all human organs through various signaling mechanisms, which are only now being understood. For instance, the gut microbiota can regulate estrogen levels, which in turn may induce the growth of sex hormone-dependent cancers, such as prostate cancer^[48]^. Microbial metabolites, such as SCFAs and tryptophan derivatives, modulate systemic inflammation, immune checkpoints, and hormone metabolism, impacting tumor growth and treatment responses^[49]^. The gut-brain axis further links gut dysbiosis to brain tumors, including gliomas, suggesting a broader role of the microbiome in systemic cancer biology^[50]^.

#### Local tissue/tumor microbiome

The presence of bacteria in tumors was reported about a century ago, as previously reviewed^[51]^. Recently, several studies have shown the presence of bacteria in different types of tumors, including lung, breast, prostate, and colorectal^[52-54]^. However, tumor microbiome characterization remains a challenge due to low biomass and contamination^[55]^. Compared with gut microbiome studies, several hurdles exist in the characterization of the tumor microbiome, due to confounding factors in sample handling and analysis. Key potential sources of these confounding factors include: (i) microbial contamination during biospecimen sampling; (ii) microbial DNA contamination during subsequent sample processing; and (iii) host DNA-related sequencing difficulties^[56,57]^. Doubt has been cast on the accuracy of some earlier tissue microbiome studies (e.g., placenta, tumors), as advances in technology have elucidated that many of the putative intra-tissue microbial sequences could be accounted for as contaminants^[58]^. Davis *et al.* introduced an R package “decontam” to rule out contaminations^[59]^. A number of strategies have been developed by our lab and others to address these hurdles at various stages of sampling, processing, and bioinformatics^[57,60-63]^.

The local tissue microbiome has been postulated to play a critical role in cancer development, particularly in organs like the breast, oral cavity, and lungs^[64]^. Studies have shown that each tissue harbors its own microbial community, with the potential to influence local immune responses and contribute to carcinogenesis. For instance, the breast tissue microbiome has been found to harbor unique bacterial populations^[63]^. Xuan *et al.*, using 16S sequencing, reported that breast tumor tissue was enriched with *Methylobacterium radiotolerans* while adjacent normal tissue was enriched with *Sphingomonas yanoikuyae*, indicating dysbiosis^[65]^. These bacteria can influence tumor growth by modulating local immune responses or by producing metabolites that promote tumorigenesis. Similarly, the oral microbiome, which includes species like *Porphyromonas gingivalis*, has been linked to oral squamous cell carcinoma (OSCC). This bacterium is believed to promote tumor development, as Wen *et al.* have shown that localization of *P. gingivalis* was associated with tumor progression and poor survival of OSCC patients^[66]^.

The relevance of both local tissue and tumor microbiomes to cancer extends beyond tumor development to treatment outcomes. Several studies have demonstrated that the presence of certain bacteria within tumor tissues can impact the efficacy of cancer therapies (see later). Thus, understanding the local tissue and tumor microbiomes not only provides insights into cancer development but also opens new avenues for therapeutic strategies aimed at manipulating the microbiome to improve cancer treatment outcomes.

Beyond the gut and tumor microbiome, organ-specific microbiota also plays a critical role in cancer occurrence. The respiratory microbiota, for example, is associated with lung cancer^[67]^, where microbial dysbiosis contributes to inflammation, immune modulation, and carcinogenesis. Similarly, the urogenital and oral microbiota have been implicated in bladder, cervical, and head and neck cancers, as previously reviewed^[68]^. These site-specific microbial communities influence local immune responses, metabolic pathways, and oncogenic signaling.

## SO WHAT?

### Microbiome in tumor progression

The microbiome’s involvement in tumor progression extends across various cancer types through its interactions with the immune system, tumor metabolism, and local tissue environments^[69]^. Certain bacteria within tumors have been found to produce metabolites that promote cancer cell proliferation. For instance, *Fusobacterium nucleatum* not only aids in immune evasion but also metabolically supports CRC progression by producing metabolites that fuel cancer cell growth^[70]^. Similarly, the breast cancer microbiome may contribute to tumor progression by altering the local tissue environment through metabolite production or direct interaction with cancer cells. Studies have shown that microbial communities within the tumor microenvironment (TME) can support angiogenesis and create a hypoxic environment favorable for tumor growth, thereby accelerating disease progression^[71-73]^. The complexity of these interactions underscores the significance of the microbiome in regulating tumor behavior, highlighting the potential for targeting the microbiome as part of therapeutic strategies in cancer treatment.

Beyond gastrointestinal and breast cancers, microbiota is implicated in the progression of lung cancer, gliomas, and urogenital cancers. The respiratory microbiota, for example, alters immune homeostasis in lung cancer, promoting inflammation and resistance to immunotherapy^[74]^. In gliomas, gut microbiota-derived metabolites such as SCFAs and tryptophan derivatives impact neuroinflammation and tumor progression^[75]^. Similarly, microbial dysbiosis in the urogenital tract influences cervical and bladder cancer development through chronic inflammation and immune modulation^[76]^.

### Clinical implication of the microbiome in relation to treatment efficacy

Certain bacteria can alter the pharmacodynamics and pharmacokinetics of therapeutic agents, either enhancing or reducing their efficacy^[54]^. For instance, *Bacteroides vulgatus* and *Bacteroides dorei* have been shown to predict immune-related adverse events associated with immune checkpoint inhibitors (ICIs)^[77]^. Gut microbial dysbiosis induced by chemotherapy, such as a reduction in beneficial bacteria like Firmicutes, can also exacerbate treatment side effects, including gastrointestinal mucositis^[78]^. Conversely, a key study in the field, performed by Baruch *et al.*, showed that fecal microbiota transplant (FMT) improved the response to immunotherapy in melanoma patients^[79]^. Griffin *et al.* have shown that the genus *Enterococcus* correlates with responses in patients treated with immune checkpoint therapy^[80]^. Dai *et al.* have reported that *Mycoplasma hyorhinis* promotes tyrosine kinase inhibitor (TKI) resistance in patients with lung adenocarcinoma^[81]^, while other studies report the dramatic decrease in cytostatic activity of gemcitabine in pancreatic cancer^[82]^. On the contrary, certain bacterial species have been associated with an enhanced response to this same drug^[54]^. Thus, the microbiome is both a potential therapeutic target and a factor influencing the effectiveness of existing cancer treatments.

### Chemotherapy

The microbiome has been shown to significantly impact the efficacy of chemotherapy, both directly by modulating drug metabolism and indirectly influencing host immune responses. Studies have demonstrated that beneficial gut bacteria like *Bifidobacterium* and *Akkermansia muciniphila* are associated with improved responses to chemotherapy, as these microbes help maintain gut barrier integrity and promote a more effective immune-mediated antitumor response^[83,84]^. Conversely, dysbiosis may lead to increased intestinal permeability and inflammation, reducing the host’s ability to respond to chemotherapy. Certain bacterial species can modify chemotherapeutic agents, thereby reducing or increasing their efficacy^[85,86]^. A study from our group examined the effects of bacterial species identified in tumor samples from breast cancer patients on 30 standard chemotherapies *in vitro.* Results demonstrated an increase in the toxicity of six chemotherapeutic drugs, a decrease in nine, including doxorubicin and gemcitabine, and no effect in the remaining 15, with different bacterial species producing different effects^[54]^. This was also verified in an *in vivo* mouse tumor model, with inhibitory effects on gemcitabine evident in tumors colonized by *E. coli*. This interaction underscores the importance of tumor-associated microbiota in directly altering drug pharmacodynamics, thereby influencing the effectiveness of chemotherapy. Beyond enzymatic activity, *Enterococcus faecalis* can produce ROS that damage DNA and promote resistance to therapy^[87]^. Evidence also suggests that chemotherapeutic agents can cause dysbiosis, leading to adverse effects such as mucositis and systemic inflammation, further complicating the treatment process^[88]^. Thus, maintaining a healthy microbiome is critical for supporting chemotherapy efficacy and minimizing side effects.

Additionally, microbial metabolites influence chemotherapy responses. For example, SCFAs produced by gut bacteria have been found to enhance the efficacy of certain chemotherapeutic drugs by promoting apoptosis in cancer cells^[89]^. SCFAs such as butyrate not only serve as energy sources for colonocytes but also regulate immune responses and influence epigenetic modifications in cancer cells^[90]^. The production of these metabolites by the gut microbiota can support chemotherapeutic efficacy by modulating tumor cell sensitivity to treatment^[91]^. This relationship suggests that manipulating the microbiome or supplementing SCFA levels could serve as a strategy to enhance chemotherapy effectiveness in certain cancer types.

### Different types of chemotherapies

#### Cisplatin

Cisplatin is used against various types of cancers at advanced stages. It has the potential to cause gut bacterial dysbiosis due to its antibacterial effects on both Gram-positive and Gram-negative bacteria^[92]^. In addition to its side effects such as weight loss and ototoxicity, possibly involving gut microbiota, it can bind to the DNA of epithelial cells, resulting in impaired replication and ultimately posing a risk to gut barrier integrity^[93]^. However, co-administration of D-methionine is used as a preventive measure against its toxicity. D-methionine helps promote the growth of *Lactobacillus* and *Lachnospiraceae*^[94]^. Moreover, the microbiome has been shown to have the potential to impact the efficacy of cisplatin. Gui *et al.* have reported a reduction in the efficacy of cisplatin in mice when administered alone compared to those co-administered with probiotics^[95]^.

In addition to SCFAs, indole derivatives, such as indole-3-lactic acid and indole-3-pyruvic acid, play a crucial role in cancer progression and therapy response^[96]^. These microbial metabolites influence the TME, modulate immune responses, and impact the efficacy of chemotherapeutic drugs by altering drug metabolism and resistance^[97]^. Their integration into oncobiome research could provide novel insights into microbiome-based cancer therapies.

#### Cyclophosphamide

Cyclophosphamide works as an immune stimulant against cancer^[98]^. Studies using murine models have reported the immune inhibition as a result of cyclophosphamide when co-administered with antibiotics specifically targeting gram-negatives, consequently reducing the efficacy of treatment^[99]^. However, this has been shown to be reversed, at least in parts, when administered with probiotics supplementation such as *Lactobacillus plantarum*^[100]^.

#### 5-Fluorouracil

5-Fluorouracil is frequently used in the treatment of gastric tumors. Its mechanism of action involves the inhibition of a key enzyme, thymidylate synthase^[101]^. Gastrointestinal effects, including mucositis, limit its usefulness as its one dose may result in gut bacterial dysbiosis. This induced dysbiosis is characterized by the reduction in beneficial bacteria such as *Lactobacillus* and an increase in *Enterococcus* and *Escherichia*^[102]^*.* A rodent trial conducted by Yuan *et al.* reported that probiotic supplementation can help improve the efficacy of 5-fluorouracil^[103]^.

#### Gemcitabine

Gemcitabine, a pyrimidine antagonist, competes with deoxycytidine in the process of DNA synthesis^[104]^. As described earlier, gemcitabine was first reported in 2015 to be inactivated through its metabolism by certain intratumoral bacteria in murine cancer models^[54]^, with similar observations since then^[105]^. Other murine studies have reported gemcitabine resistance due to its metabolic conversion into difluoro-deoxy-uridine, which happens as a result of the active participation of the bacterial enzyme cytidine deaminase^[106]^. This enzyme is primarily reported in Gammaproteobacteria^[107]^. However, co-administration of antibiotics like ciprofloxacin happens to improve the antitumor potential of gemcitabine^[108]^.

#### Irinotecan

Irinotecan, a DNA replication inhibitor, and its active form SN-38 are effective against various types of cancer^[109]^. It is important to note that this drug is excreted in the intestine in its inactive form (SN-38G), where an enzyme (ß-glucuronidase) produced by *E. coli* converts it back to its active form^[110]^. Consequently, this process results in enteric injury, causing diarrhea^[111]^. However, these toxic side effects can be prevented by administering ß-glucuronidase inhibitors^[112]^.

#### Immunotherapy

**Figure 2:**
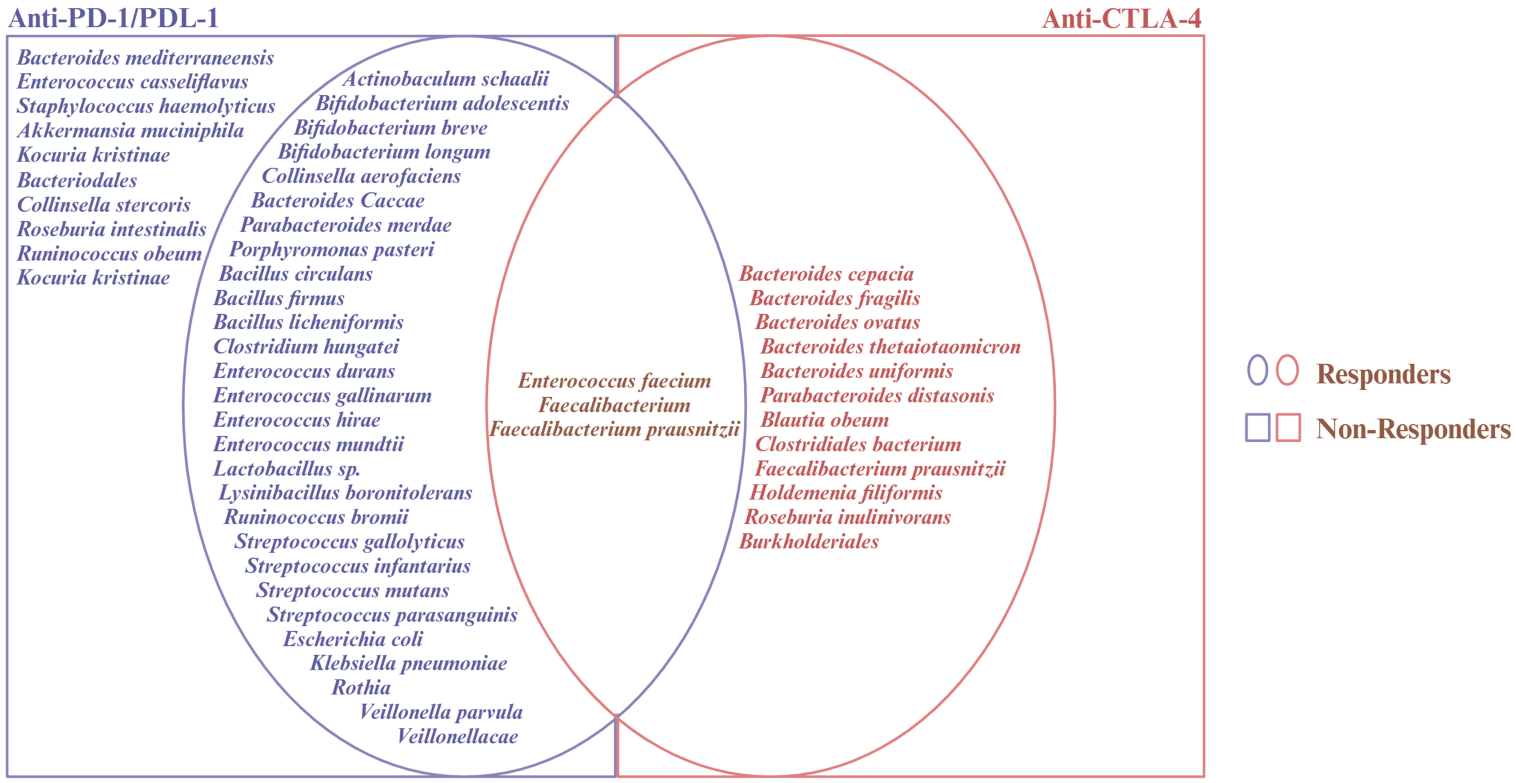
Microbial enrichment during immunotherapy. This figure illustrates the effects of different members of the gastrointestinal microbiota on ICI treatments. The studies referenced are by Temraz *et al.*^[119]^, Frankel *et al.*^[120]^, Routy *et al.*^[121]^, Chaput *et al.*^[122]^, and Matson *et al.*^[123]^. ICI: Immune checkpoint inhibitor.

Currently, cancer immunotherapy primarily involves the administration of ICI monoclonal antibodies, including anti-programmed cell death-1 (anti-PD-1), anti-programmed cell death ligand-1 (anti-PDL-1), anti-cytotoxic T lymphocyte-associated protein-4 (anti-CTLA-4)^[113]^. The gut microbiome has been reported to influence such immunotherapy; for example, high abundances of *Bifidobacterium* and *Akkermansia muciniphila* in the gut have been shown to be associated with better response to anti-PD-1 and anti-CTLA-4 treatments, respectively^[114,115]^. This is due to the infiltration of cytotoxic T cells (a critical step in cancer) promoted by these bacteria. Conversely, gut bacterial dysbiosis has been linked to immunotherapy resistance^[116]^. However, clinical studies, such as that by Davar *et al.*, have shown that FMT from responders to non-responsive melanoma patients restored sensitivity to immunotherapy, particularly anti-PD-1, leading to tumor regression^[117]^; similar findings have been reported by Routy *et al.* in a phase-1 trial^[118]^ (see Figure 2 for the association between microbiota and responders and non-responders). Such clinical findings emphasize the direct role of the gut microbiome in modulating immunotherapy response, suggesting microbiome-driven interventions could be a promising strategy to overcome immunotherapy resistance.

Besides enhancing ICI response, microbiota activity seems to have the potential to overcome the associated immune-related adverse events, such as colitis, which are linked to alterations in the gut microbiota^[124]^. Recently, Gao *et al.* have shown that mice administered with *Faecalibacterium prausnitzii* experienced fewer immune-related side effects during dual anti-PD-1 and anti-CTLA-4 therapy^[125]^. The fact that *Faecalibacterium prausnitzii* increased alpha diversity and improved the abundance of beneficial bacteria suggests that maintaining a balanced gut microbiome could help mitigate the toxicity of immunotherapies, improving both efficacy and tolerability.

### Radiotherapy

The microbiome modulates the effects of radiotherapy, particularly through its influence on immune responses and the body’s ability to repair radiation-induced damage^[126]^. A murine model study revealed that the indigenous gut bacterial species *Lactobacillus acidophilus* was involved in the recovery of irradiation-induced intestinal damage^[127]^. These bacteria promote the production of anti-inflammatory cytokines and reduce oxidative stress, helping to protect healthy tissues from radiation-induced damage while simultaneously promoting tumor destruction^[128]^.

In contrast, dysbiosis can exacerbate the side effects of radiotherapy by increasing systemic inflammation and compromising gut barrier function. Radiation-induced mucositis, a common side effect, has been linked to changes in the gut microbiota that promote inflammation and damage to the gastrointestinal lining, as shown by Segers *et al.*^[129]^. This not only affects the patient’s quality of life, but also limits the dosage of radiation that can be safely administered^[130,131]^. Studies have shown that probiotic supplementation during radiotherapy can reduce the severity of mucositis and support recovery by restoring gut microbiota balance, suggesting a potential avenue for improving radiotherapy tolerance through microbiome modulation^[132,133]^.

## NOW WHAT?

### Microbiome modulation

The intricate relationship between systemic lymphoid tissues and gut microbiota suggests the potential for microbial modulation in cancer therapy. Preliminary research indicates that intratumoral microbiota could be present in many tumor types and have a significant impact on immune and other responses^[134]^. This relationship necessitates considering microbial niches and their interactions, as changes in gut microbiota may impact both the gastrointestinal and intratumoral microbiomes, potentially influencing treatment outcomes^[135]^. However, the effects can be inconsistent; for example, the modulation of gut microbiota was shown to influence tumor microbiota in pancreatic cancer, perhaps via the pancreatic duct^[136]^. On the other hand, gut microbiota is negatively affected by antibiotics, reducing treatment efficacy, such as chemotherapy^[137]^. Given the complexities of this interface, in-depth studies are required to gain a mechanistic understanding and improve clinical assessments, ultimately making the use of probiotics, prebiotics, or antibiotics a viable option in cancer treatment.

Direct regulation of intratumoral microbiota is an emerging area, with approaches such as engineered bacteria designed to selectively colonize tumors^[138]^, targeted antibiotic or phage therapy to deplete tumor-promoting microbes, and microbiome-based metabolic interventions to reshape the TME^[139]^. While still in the early stages, these strategies highlight the potential for microbiome-directed cancer therapies. Further research is needed to refine these techniques for precision oncology applications.

### Diet

Nutrition interventions may play an important role in managing cancer alongside medical treatment. Dietary strategies incorporate bioactive compounds important to overall health, cancer risk, post-treatment outcomes, and overall survival. For example, a growing body of evidence suggests that along with weight management, a high intake of fruits, vegetables, whole grains, fish, and poultry may protect against cancer^[140,141]^. Conversely, a high intake of western diets rich in starches, red meat, alcohol, and fats is often associated with an increased risk of cancer, due to higher bile acid accumulation and reduced short-chain SCFA f by gut bacteria. This imbalance can foster an environment conducive to CRC development^[142]^. Conversely, diets rich in fiber can promote the production of beneficial SCFAs like butyrate^[143]^, which has been shown in some studies to enhance the efficacy of certain cancer treatments, such as irinotecan, by improving the drug’s activation within cancer cells^[144]^.

Animal studies have begun to explore how diet can modulate the efficacy of cancer therapies. Diets supplemented with proteins like casein or whey, L-leucine, fish oil, and oligosaccharides have demonstrated the ability to prevent bacterial translocation in chemotherapy-induced neutropenia, as previously reviewed, suggesting a protective role against treatment toxicity^[145]^. Similarly, the consumption of dietary fibers like inulin and fructooligosaccharides (FOS) has been linked to reduced toxicity from irinotecan by promoting butyrate production in the gut, which might also enhance the drug’s antitumor effects^[146]^. However, these findings need careful interpretation in humans, especially considering the complex nutritional needs of cancer patients who might be malnourished or have undergone treatments affecting their digestive system.

Clinical research into dietary interventions also includes fasting-mimicking diets (FMD), which have shown potential in improving cancer treatment outcomes by modulating the immune system^[147]^. FMD has been observed to reduce immunosuppressive cells while boosting cytotoxic T cells and natural killer (NK) cells, which could improve the body’s response to cancer^[148]^. Additionally, in patients with chronic myeloid leukemia, fasting diets have been associated with reduced levels of certain blood cells and leukemia-related transcripts, suggesting a possible role in managing the disease alongside conventional treatments^[149]^.

Molecular pathological epidemiology (MPE) provides a powerful framework for investigating the interaction between lifestyle, diet, environmental exposures, tumor molecular characteristics, and microbiota in cancer development and progression^[150]^. MPE studies can identify microbial signatures linked to specific dietary patterns and tumor subtypes, providing insights into how personalized nutrition and lifestyle interventions may modulate the TME^[151]^. Integrating MPE into oncobiome research will advance precision oncology and prevention, offering novel strategies for targeted cancer management based on the interplay between host genetics, microbial ecosystems, and external factors.

### Dietary supplements

The interaction between dietary supplements and the gut microbiome presents another avenue for influencing cancer treatment outcomes. For instance, jujube powder has been shown to enhance CD8^+^ T cell presence in mouse models of colon cancer^[152]^, potentially by altering the gut bacteria composition to favor butyrate-producing species. Similarly, ginseng has been explored for its ability to potentiate chemotherapy effects, though results are inconsistent across different studies. Ellagic acid, found in certain fruits, interacts with gut bacteria to produce urolithins, which might enhance chemotherapy sensitivity in colon cancer cells^[153]^. These examples highlight the complex interplay between diet, microbiota, and cancer therapy, indicating a need for more focused clinical research to translate these findings into practical therapeutic strategies.

### Probiotics and prebiotics

Probiotics, which are live microorganisms that confer health benefits to the host^[154]^, have gained attention for their potential role in modulating gut microbiota to combat cancer^[140]^. Probiotic supplementation has been shown to restore a healthy gut microbial balance^[155]^, enhance immune function, and reduce inflammation, thereby creating an environment that is less conducive to cancer initiation. For instance, several studies have demonstrated that certain probiotics, such as members of *Lactobacillus* and *Bifidobacterium* genera, can inhibit the growth of harmful bacteria and reduce the production of carcinogenic metabolites, leading to a reduced risk of CRC^[156-158]^. Additionally, probiotics have been observed to stimulate the production of anti-inflammatory cytokines and enhance the activity of immune cells, such as NK cells, that target and destroy cancerous cells^[159]^. In the context of cancer treatment, probiotics have shown potential in mitigating the side effects of cancer therapies, such as chemotherapy, radiotherapy, and immunotherapy, which can disrupt the gut microbiota and cause gastrointestinal toxicity^[160]^. Thus, probiotics can help maintain the integrity of the intestinal barrier and prevent opportunistic infections, improving the patient’s overall tolerance to cancer treatments. Prebiotics, which foster beneficial gut microbes, have shown promise in preclinical cancer models by enhancing antitumor immunity and therapy outcomes, leading to clinical trials^[161,162]^. However, the field lacks a comprehensive understanding of dietary impacts on cancer due to challenges in data collection. Postbiotics, microbial by-products, are less studied but offer potential benefits due to their defined nature^[163]^. FMT has proven effective for conditions like *Clostridioides difficile* infection and shows some benefits in managing immunotherapy-related colitis, although its long-term effects are still under investigation^[164,165]^. While more research is needed to fully understand the therapeutic potential of probiotics and prebiotics in oncology, current findings highlight their promising role in supporting gut health and augmenting cancer prevention and treatment strategies.

### Antibiotics in cancer

Antimicrobial therapy in cancer treatment primarily focuses on addressing infections from known microbial carcinogens like *H. pylori* and specific viruses^[166]^. For instance, gastric lymphomas linked to *H. pylori* are treated with antibiotic regimens. However, the application of antibiotics in treating solid tumors like lung, colon, and pancreatic cancers^[167]^ has shown mixed results; some studies suggest benefits like reducing inflammation or enhancing immune responses^[168]^, while systemic antibiotics can negatively impact the efficacy of ICIs and patient survival^[169]^. In hematologic cancers, a delicate balance exists where antibiotics might either prevent or promote leukemic progression in genetically susceptible individuals^[170]^.

### Microbiome as a biomarker for precision oncology

The microbiome has emerged as a promising biomarker for precision oncology due to its influence on cancer and treatment outcomes. A number of studies have demonstrated that specific microbial signatures correlate with distinct cancer types and stages. For example, the microbiome profile of breast tumor biopsy has been shown to indicate malignancy status^[63]^. The ability to similarly match gut bacterial profiles via stool “biopsy” would offer the potential for non-invasive diagnostics or treatment decision support. For instance, *H. pylori* infection is a well-established biomarker for gastric cancer, where chronic inflammation driven by the bacteria predisposes individuals to malignancy^[171]^. In addition to the presence of a specific bacterium, overall shifts in gut microbial profiles have also been discussed as non-invasive diagnostics, such as *Fusobacterium* for CRC^[172]^.

**Figure 3:**
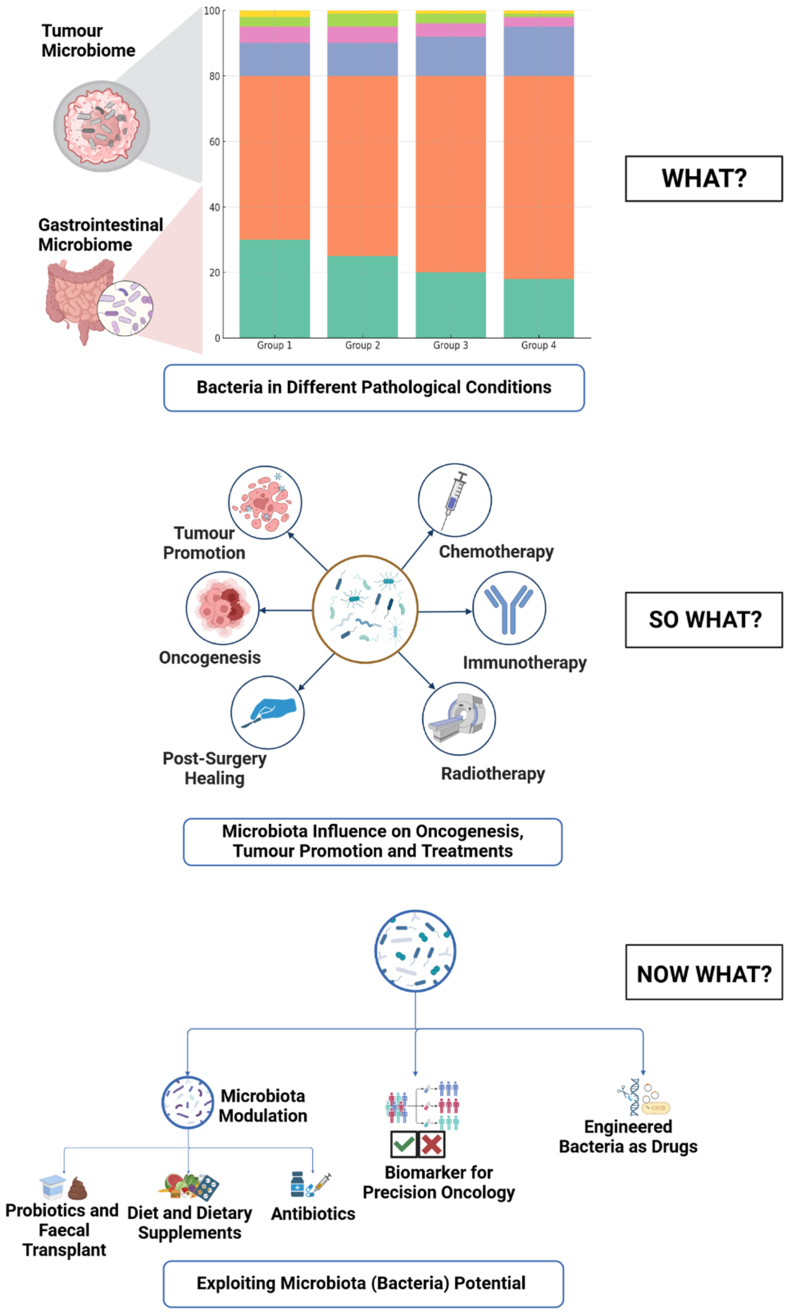
This schematic summarizes the role of microbiota in cancer and therapeutic outcomes, presented in three panels: What? The left panel highlights bacterial enrichment in different pathological conditions, such as in responders and non-responders to immune checkpoint therapies. So What? The middle panel illustrates how microbiota influences various aspects of cancer biology, including tumor promotion, oncogenesis, post-surgery healing, and responses to chemotherapy, immunotherapy, and radiotherapy. Now What? The right panel outlines potential applications of microbiota in cancer management, including modulation, use of microbiota as biomarkers for precision oncology, and engineering bacteria as therapeutic drugs.

More recent studies using 16S rRNA sequencing and metagenomics have identified distinct microbial profiles that could predict not only cancer occurrence but also patient response to therapies, supporting the use of the microbiome in personalizing cancer treatment strategies [Figure 3]. Specific bacterial species have been reported to predict therapeutic efficacies, suggesting that the composition of an individual’s microbiome could serve as a valuable biomarker for tailoring cancer treatments. Zhao *et al*. have shown the role of the gut microbiome, *Bifidobacterium breve* in particular, in predicting the efficacy of combined chemotherapy and immune therapy (anti-PD-1) in patients with non-small cell lung cancer (NSCLC). For responders, they reported a strong association between *B. breve* and enhanced progression-free survival^[173]^. Similarly, Liu *et al.* demonstrated that patients with higher abundances of fecal *Faecalibacterium* and *Lachnospiraceae* had only mild immune-related adverse events during anti-PD1 therapy compared with those having severe adverse events and presented a higher abundance of *Streptococcus*, *Paecalibacterium*, and *Stenotrophomonas*^[174]^*.* This suggests that modulating the microbiome could enhance therapeutic efficacy or reduce adverse effects, positioning microbial profiling as a powerful tool in precision oncology to tailor treatment based on individual microbiota phenotypes.

In addition to microbial composition, microbial extracellular vesicles (EVs) have emerged as significant biomarkers, as previously reviewed^[175]^. These EVs, found in both blood and tumor tissues, carry microbial-derived molecules that influence cancer progression and immune modulation^[176]^. Notably, the profiles of microbial EVs exhibit distinct differences between cancer patients and healthy individuals, underscoring their potential in assessing cancer risk, disease progression, and treatment response^[177]^. Their integration into microbiome-based diagnostics could enhance precision oncology by providing non-invasive and dynamic biomarkers for monitoring cancer.

Recent advancements in microbiome-based biomarkers have expanded the potential for precision oncology. A 2023 study utilized engineered bacteria to detect tumor-specific DNA in CRC^[178]^, representing a significant leap in how genetic engineering can improve cancer diagnostics. In addition to fecal samples, other body fluids, including blood, urine, cerebrospinal fluid, gastric juice, and lung fluid, have been identified as sources for detecting microbial components, enabling non-invasive cancer diagnosis and prognosis evaluation across different tumor types^[179]^. These developments highlight the growing versatility of microbiome-based biomarkers in personalized cancer care.

### Engineered bacteria as cancer drugs

Bacteria naturally exhibit preferential replication within tumors, often accumulating to significantly higher numbers in tumors than in healthy tissues^[180]^. This property has been harnessed to develop cancer therapies where bacteria serve as carriers for cytotoxic agents directly to cancer cells^[181-184]^. The advent of synthetic biology has enabled the engineering of microbes as novel cancer therapies. This cutting-edge approach leverages the natural ability of bacteria to target and colonize tumors^[185]^. Genetically modified non-pathogenic bacteria, such as *E. coli* and *Salmonella*, selectively proliferate in the immune-privileged and hypoxic TME, where they can produce or deliver therapeutic payloads such as immune-stimulating molecules, enzymes, or nucleic acid^[186,187]^.

Bacterial strategies can target different “compartments” within tumors. In addition to malignant cells (targeted by invasive bacteria) and tumor stroma (targeted by non-invasive bacteria), intratumoral immune cells can also be targeted. For example, our group has reported a phagocytic cell-selective strategy^[188]^.

By harnessing the ability of engineered microbes to penetrate deep into tumor tissue, researchers have designed bacteria to produce anti-cancer compounds in response to specific environmental cues within tumors. *Clostridium* spores have been examined at preclinical and clinical levels for several decades. Janku *et al.* have shown that injected spores germinate in the anaerobic core of solid tumor, resulting in a transient systemic cytokine response and enhanced systemic tumor-specific T cell responses^[189]^. Chowdhury *et al*. demonstrated that engineered bacteria could produce and release an antibody against CD47, stimulating an immune response against tumors, including those not directly injected, without causing common side effects^[190]^. Similarly, a study by Wu *et al.* in 2022 reported the macrophage-mediated tumor-targeted delivery of engineered *Salmonella typhimurium* VNP20009, which expressed and secreted anti-PD-1 nanobodies that promoted tumor regression and reduced associated toxic effects in a mouse model of melanoma^[191]^. These bacteria not only inhibit tumor growth but also activate immune responses, enhancing their therapeutic potential as both direct anti-cancer agents and immune adjuvants. The TME can also be “conditioned” by bacteria to improve other therapies, such as oncolytic viral therapy as demonstrated by our group^[192]^.

The concept of using bacteria as programmable therapeutic agents could potentially be tailored to individual patients or specific tumor types, significantly expanding treatment options for cancer with reduced toxicity and fewer side effects. However, potential risks must be carefully considered. Safety concerns include uncontrolled bacterial growth, systemic infections, and off-target effects, which could lead to adverse immune reactions^[193]^. Additionally, the risk of horizontal gene transfer raises concerns about spreading engineered genetic material to commensal microbiota. Strategies such as genetic kill switches, antibiotic sensitivity controls, and precise dosing are being developed to mitigate these risks^[194]^. A thorough understanding of these challenges is essential for the safe clinical translation of engineered bacteria in oncology.

## LIMITATIONS

It is important to note that some of the studies referenced in this review are based on relatively small sample sizes. This may limit the generalizability of their findings and should be interpreted with caution. Future large-scale, multicentre studies are necessary to validate these results and confirm the clinical relevance of microbiome-based approaches in cancer research and therapy.

## CONCLUSION

The relationship between the microbiome and cancer is increasingly recognized as a significant factor in cancer development and progression. Dysbiosis in microbial communities contributes to oncogenesis by promoting chronic inflammation, modulating immune responses, and altering the TME. The gut microbiota, bacteria in particular, are linked to cancer types such as colorectal, gastric, and liver cancers, while local microbiomes in tissues like the breast and oral cavity are also implicated in carcinogenesis. The role of the microbiome extends beyond oncogenesis to influence disease progression and treatment outcomes. Microbial composition affects the efficacy and toxicity of chemotherapy, radiotherapy, and immunotherapy. Certain bacterial species can enhance drug responses, while others may lead to resistance or adverse effects. As a result, while the microbiome can be modulated via dietary practices and the use of prebiotics and probiotics, it is emerging as a valuable biomarker for precision oncology, offering personalized treatment strategies based on microbial profiling. In addition, engineered microbes hold promise as a novel cancer therapy designed to selectively target tumors and produce therapeutic agents. This approach aims to improve treatment precision and reduce systemic toxicity. Overall, the integration of microbiome research into oncology is transforming how we understand and approach cancer treatment, offering new opportunities for diagnosis, therapy, and improved patient outcomes.

## DECLARATIONS

### Copyright

© The Author(s) 2025.
